# Infants and toddlers with sensitization to peanut are often co‐sensitized to tree nuts

**DOI:** 10.1002/clt2.70008

**Published:** 2024-11-05

**Authors:** Lara Meixner, Stephanie Heller, Friederike Bluhme, Valérie Trendelenburg, Kirsten Beyer, Birgit Kalb

**Affiliations:** ^1^ Department of Pediatric Respiratory Medicine, Immunology and Critical Care Medicine Charité—Universitätsmedizin Berlin, Corporate Member of Freie Universität Berlin and Humboldt‐Universität zu Berlin Berlin Germany

**Keywords:** eczema, food allergy, infants, peanut sensitization, toddlers, tree nut sensitization

To the Editor,

Especially infants with eczema are at high risk for developing food allergies and it is the current understanding that sensitization occurs via the cutaneous route due to an impaired skin barrier function.^1^, ^2^ Accordingly, a high peanut consumption in the household has been shown to be a possible risk factor for developing peanut allergy in infancy.^3^ Therefore, German S3‐guidelines on allergy prevention recommend that peanut allergy should first be ruled out in infants with moderate to severe atopic dermatitis, before introducing peanut into the infant's diet for preventive purposes.^4^ During the last decades, vegan and plant‐based diets have become a growing trend.^5^ Tree nuts, such as cashews, hazelnuts and walnuts are a nutritional mainstay of plant‐based diets and plant‐based alternatives for milk and milk‐products often contain tree nuts.^5^ These changes in dietary habits may lead to a wider spread of tree nut allergens in households, increasing the risk for cutaneous exposure. There are hints, that individuals with peanut allergy have a higher likelihood of being allergic to tree nuts compared to the general population.^6^, ^7^ Therefore, the aim of this study was to investigate how often peanut‐sensitized infants and toddlers are sensitized to cashew, hazelnut and walnut as well as their seed storage proteins, which might be associated with a high risk for clinical reactivity.

The study cohort consists of infants and toddlers who were referred to the Department of Pediatric Respiratory Medicine, Immunology and Critical Care Medicine, Charité—Universitätsmedizin Berlin. Some of the patients underwent an oral food challenge (OFC) for routine diagnostics between 2007 and 2020. Blood as well as clinical data was collected from all patients in the frame of routine diagnostics. Inclusion criteria for the analysis of co‐sensitization was age ≤2 years and specific IgE (sIgE) to peanut ≥0.1 kU/l.

The detection of sIgE to peanut, hazelnut, walnut and cashew and to their respective 2S albumins Ara h 2, Cor a 14, Jug r 1, Ana o 3 as well as to the 7S vicilin‐like globulin Ara h 1, was performed by using the NOVEOS^TM^ immunoanalyzer (Garden Grove, California, USA). Sensitization was defined as sIgE ≥0.1 kU/l.

In order to determine the probability for a positive hazelnut food challenge by Cor a 14‐sIgE and for a positive cashew food challenge by Ana o 3‐sIgE for each patient, probability curves by Beyer et al. and Lange et al. were utilized.^8^, ^9^ Since there is no probability curve available for walnut, the individual risk for a positive OFC with walnut could not be estimated (see Supporting Information S1 for detailed methods).

Sera from 101 peanut‐sensitized patients (peanut‐sIgE ≥0.1 kU/l) were analyzed. The median age of the patients at the time of blood drawing was 16 months (range: 5–24 months). Nearly all patients (98%) suffered from eczema. Details on patient characteristics are provided in Table 1.

**TABLE 1:** Characteristics of the study population.

	All *n* = 101 (100%)	Hazelnut sensitized *n* = 95 (94.1%)	Walnut sensitized *n* = 88 (87.1%)	Cashew sensitized *n* = 85 (84.2%)
Gender male (*n* (%))	68 (71.6)^a^	67 (72.8)^a^	65 (74.7)^a^	62 (72.9)^a^
Age in months, median (range)	16 (5–24)	16 (5–24)	16 (5–24)	16 (5–24)
Eczema (*n* (%))	99 (98.0)	93 (97.9)	86 (97.9)	83 (97.7)
sIgE to peanut in kU/l, median (range)	1.23 (0.10–75.93)	1.17 (0.10–75.93)	1.08 (0.10–75.93)	1.23 (0.10–75.93)
sIgE to Ara h 1 (*n* (%))	84 (83.2)	81 (85.3)	75 (85.2)	74 (87.1)
sIgE to Ara h 1 in kU/l, median (range)	0.38 (0.10–12.64)	0.40 (0.10–12.64)	0.43 (0.10–12.64)	0.40 (0.10–12.64)
sIgE to Ara h 2 (*n* (%))	71 (70.3)	67 (70.5)	60 (68.2)	60 (70.6)
sIgE to Ara h 2 in kU/l, median (range)	0.98 (0.10–43.94)	0.80 (0.10–43.94)	0.69 (0.10–43.94)	0.78 (0.10–43.94)
Oral food challenge proven peanut allergy (*n* (%))	33 (40.7)^b^	31 (39.2)^b^	26 (35.6)^b^	27 (38.03)^b^
sIgE to extract^c^ in kU/l, median (range)	‐	1.21 (0.11–101)	0.87 (0.10–101)	0.97 (0.10–101)
sIgE to 2S‐albumin^d^ (*n* (%))	‐	42 (41.6)	42 (41.6)^e^	40 (39.6)^e^
sIgE to 2S‐albumin^d^ in kU/l, median (range)	‐	0.39 (0.10–101)	0.41 (0.10–101)	0.46 (0.10–101)

Abbreviations: kU/l, kilounits/liter; sIgE, specific immunoglobulin E.

^a^ data from *n* = 95 (all), *n* = 92 (hazelnut sensitized), *n* = 87 (walnut sensitized), *n* = 85 (cashew sensitized).

^b^ data from *n* = 81 (all), *n* = 79 (hazelnut sensitized), *n* = 73 (walnut sensitized), *n* = 71 (cashew sensitized).

^c^ hazelnut, walnut or cashew extract.

^d^ hazelnut: Cor a 14; walnut: Jug r 1; cashew: Ana o 3.

^e^ one patient was sensitized only to the 2S albumin, not to the extract.

Specific IgE ≥0.1 kU/l to at least one tree nut was detected in 96.0% (*n* = 97) of the patients. Most children were sensitized to hazelnut (*n* = 95; 94.1%), followed by walnut (*n* = 88; 87.1%) and cashew (*n* = 85; 84.2%). More than a half (59/101; 58.4%) were sensitized to at least one 2S albumin. 42 patients (41.6%) were sensitized to Cor a 14 and Jug r 1, while 40 patients (39.6%) were sensitized to Ana o 3 (sIgE levels are provided in Table 1). We detected sIgE ≥0.1 kU/l to all three tree nuts in 80.2% (*n* = 81). Furthermore, 26.7% (*n* = 27) of the children showed sIgE to all of the corresponding 2S albumins. Children sensitized to all three 2S albumins tended to have higher sIgE levels to the extracts than those sensitized to none or one 2S albumin (data not shown). Of the participants aged ≤12 months (*n* = 26; 25.7%), 88.5% (*n* = 23) were sensitized to at least one tree nut, 46.2% (*n* = 12) to at least one 2S albumin and 34.6% (*n* = 9) to all three 2S albumins (data not shown).

Five of the 101 peanut‐sensitized infants and toddlers (5.0%) would have reacted with 90% probability to hazelnut and 14 (13.9%) to cashew. In total, 15.8% (*n* = 16) of all peanut‐sensitized children had an at least 90% predicted probability to be allergic to hazelnut and/or cashew (Figure 1).

**FIGURE 1:**
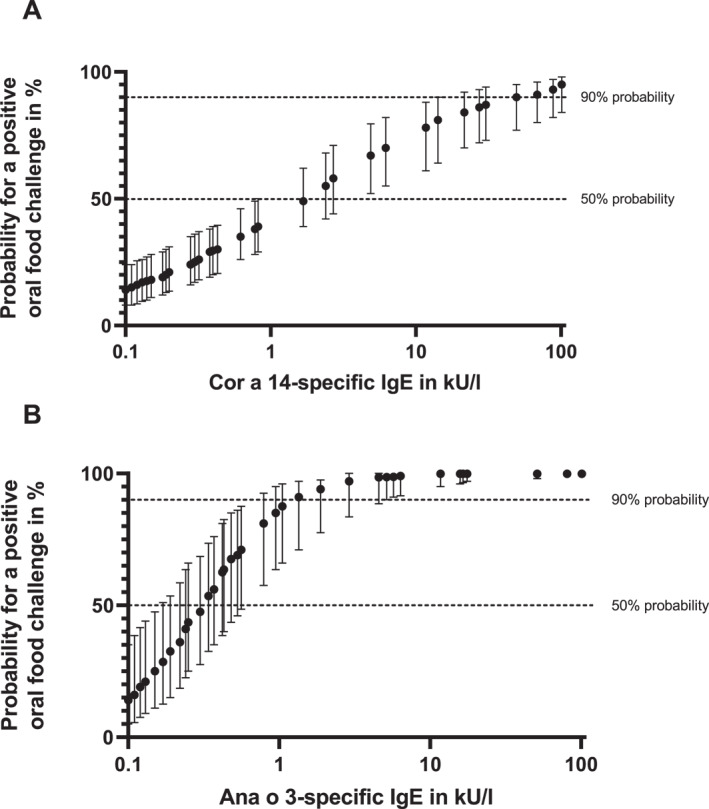
Predicted values for an allergic reaction to hazelnut (A) and cashew (B) for each patient. Values were estimated according to the patient's individual Cor a 14‐sIgE and Ana o 3‐sIgE values. Those were compared to a given probability for a positive OFC outcome at a certain sIgE value outlined in the prediction curves for Cor a 14 by Beyer et al.^8^ and for Ana o 3 by Lange et al.,^9^ respectively.

We were able to show that peanut‐sensitized infants and toddlers are often co‐sensitized to tree nuts and their 2S albumins. Regarding co‐existing tree nut allergy among peanut‐allergic children several observational studies showed that peanut allergy is a risk factor for tree nut allergy.^6^, ^7^ Moreover, our data indicates that even very young peanut‐sensitized children with eczema below 2 years of age may frequently be sensitized to tree nuts and their seed storage proteins. Almost all of the children included in our analysis (98%) suffered from eczema, which is known to be a major risk factor for sensitization and the development of food allergy.^2^

A few limitations of our study have to be mentioned. As this was a retrospective analysis of stored serum samples, we had no data on the clinical relevance of the tree nut sensitization. In particular, we lacked information on whether an oral food challenge had been conducted or what its outcome was. Moreover, the period of study inclusion was relatively long (2007–2020). However, specific recommendations for the early introduction of food allergens (e.g. peanut) for allergy prevention were published after this period.

In conclusion, our study at a tertiary care clinic demonstrates that a very high proportion of peanut‐sensitized infants and toddlers with eczema are already co‐sensitized to tree nuts, with a high likelihood for allergic reactions in a considerable proportion of them. Therefore, it should be considered to determine sIgE to tree nuts in peanut‐sensitized children if tree nuts have not been consumed so far. In case of a sensitization to tree nuts, an OFC should be performed to determine its clinical relevance.

## AUTHOR CONTRIBUTIONS

**Lara Meixner**: Investigation (equal); formal analysis (lead); validation (equal); writing—original draft (lead); writing—review and editing (equal). **Stephanie Heller**: Investigation (equal); validation (equal); writing—review and editing (equal). **Friederike Bluhme**: Investigation (equal); writing—review and editing (equal). **Valérie Trendelenburg**: Investigation (equal); writing—review and editing (equal). **Kirsten Beyer**: Conceptualization (equal); formal analysis (supporting); writing—original draft (supporting); writing—review and editing (lead); funding acquisition (equal); supervision (equal). **Birgit Kalb**: Conceptualization (equal); formal analysis (supporting); writing—original draft (supporting); writing—review and editing (lead); funding acquisition (equal); supervision (equal).

## CONFLICT OF INTEREST STATEMENT

Kirsten Beyer reports advisory board/consulting fees or speakers bureau from Aimmune Therapeutics, Bencard, Danone/Nutricia, DBV, Hycor, Infectopharm, Mabylon, Meda Pharma/Mylan, Nestle, Novartis and ThermoFisher as well as research grants from Aimmune, ALK, Danone/Nutricia, DBV Technologies, Hipp, Hycor, Infectopharm and Novartis outside the submitted work. Birgit Kalb reports advisory board/consulting fees from Viatris. Valérie Trendelenburg received speaker's fees from Nutricia/Danone. Friederike Bluhme, Stephanie Heller and Lara Meixner have nothing to disclose.

## Supporting information

Figure S1

## References

[1] Lack G . Update on risk factors for food allergy. J Allergy Clin Immunol. 2012;129(5):1187‐1197. 10.1016/j.jaci.2012.02.036 22464642

[2] Christensen MO , Barakji YA , Loft N , et al. Prevalence of and association between atopic dermatitis and food sensitivity, food allergy and challenge‐proven food allergy: a systematic review and meta‐analysis. J Eur Acad Dermatol Venereol. 2023;37(5):984‐1003. 10.1111/jdv.18919 36695076

[3] Fox AT , Sasieni P , du Toit G , Syed H , Lack G . Household peanut consumption as a risk factor for the development of peanut allergy. J Allergy Clin Immunol. 2009;123(2):417‐423. 10.1016/j.jaci.2008.12.014 19203660

[4] Kopp MV , Muche‐Borowski C , Abou‐Dakn M , et al. S3 guideline allergy prevention. Allergol Select. 2022;6(01):61‐97. 10.5414/alx02303e 35274076 PMC8905073

[5] Reese I , Schäfer C , Ballmer‐Weber B , et al. Vegan diets from an allergy point of view ‐ position paper of the DGAKI working group on food allergy. Allergol Select. 2023;7(1):57‐83. 10.5414/alx02400e 37056444 PMC10088878

[6] McWilliam V , Peters R , Tang MLK , et al. Patterns of tree nut sensitization and allergy in the first 6 years of life in a population‐based cohort. J Allergy Clin Immunol. 2019;143(2):644‐650.e5. 10.1016/j.jaci.2018.07.038 30171872

[7] Sasaki M , Koplin JJ , Dharmage SC , et al. Prevalence of clinic‐defined food allergy in early adolescence: the SchoolNuts study. J Allergy Clin Immunol. 2018;141(1):391‐398.e4. 10.1016/j.jaci.2017.05.041 28755784

[8] Beyer K , Grabenhenrich L , Härtl M , et al. Predictive values of component‐specific IgE for the outcome of peanut and hazelnut food challenges in children. Allergy. 2015;70(1):90‐98. 10.1111/all.12530 25308885

[9] Lange L , Lasota L , Finger A , et al. Ana o 3‐specific IgE is a good predictor for clinically relevant cashew allergy in children. Allergy. 2017;72(4):598‐603. 10.1111/all.13050 27644013

