# Supplementary material for: Infants and toddlers with sensitization to peanut are often co‐sensitized to tree nuts
Source: Clin Transl Allergy. 2024 Nov 5;14(11):e70008. doi: 10.1002/clt2.70008 (PMC11538028; doi:10.1002/clt2.70008)
Supplement: Supplementary file 1 — Figure S1 [file CLT2-14-e70008-s001.docx]

#### Supporting Information

### **Detailed explanation of methods**

### Oral Food Challenges (OFC)

OFCs were performed either in an open (6.2%) or double-blind, placebo-controlled (93.8%) manner (DBPCFC) by trained staff. The challenges were preferably conducted as DBPCFC, but in exceptional cases, they were performed in an open manner at the discretion of the physician. Before starting the OFC, patients underwent a physical examination, including an evaluation of the presence and severity of atopic dermatitis. Patients were eligible for the OFC if there were no conditions that might have affected safety (e.g. infectious diseases). Following the PRACTALL criteria [[1](#_ENREF_1)], the food challenges were stopped and rated as positive if objective allergic immediate type reactions were observed. Allergens were blinded in a food matrix (e.g. apple sauce) and fed in seven increasing doses (amounts equal to 3 mg, 10 mg, 30 mg, 100 mg, 300 mg, 1 g and 3 g of peanut protein) over 30-minutes intervals under clinical supervision. A positive challenge outcome was defined by objective clinical reactions (e.g. urticaria/angioedema, vomiting, wheezing/stridor, rhinitis/conjunctivitis and/or a decrease in blood pressure). After completion of all 7 titration steps without the occurrence of any kind of objective allergic reaction, patients received a subsequent cumulative dose of 4.5 g of peanut on the following day, proving clinical tolerance in case of no objective symptoms.

**Statistical Analysis**

All statistical analyses were performed using R version 4.2.0 (R Core Team 2022). Categorical data are shown as absolute and relative frequencies; continuous data are expressed as median and range. To test if there were significant differences in sensitization patterns between patients that were allergic or tolerant to peanut, chi-squared tests were used (key basic assumptions of the test were not fulfilled regarding sensitization to hazelnut and walnut). Wilcoxon tests were performed to analyze if there were significant differences in sIgE levels between peanut-allergic and peanut-tolerant patients. P-Values were adjusted using the Bonferroni adjustment. A p-value of < 0.05 was considered to indicate a significant difference. In order to determine the probability for a positive hazelnut food challenge by Cor a 14‐sIgE and for a positive cashew food challenge by Ana o 3‐sIgE for each patient, probability curves by Beyer et al. and Lange et al. were utilized [[2](#_ENREF_2), [3](#_ENREF_3)]. Since there is no probability curve available for walnut, the individual risk for a positive OFC with walnut could not be estimated.

1. Sampson HA, Gerth van Wijk R, Bindslev-Jensen C, et al. Standardizing double-blind, placebo-controlled oral food challenges: American Academy of Allergy, Asthma & Immunology-European Academy of Allergy and Clinical Immunology PRACTALL consensus report. J Allergy Clin Immunol. 2012;130(6):1260-74.

2. Beyer K, Grabenhenrich L, Härtl M, et al. Predictive values of component-specific IgE for the outcome of peanut and hazelnut food challenges in children. Allergy. 2015;70(1):90-8.

3. Lange L, Lasota L, Finger A, et al. Ana o 3-specific IgE is a good predictor for clinically relevant cashew allergy in children. Allergy. 2017;72(4):598-60


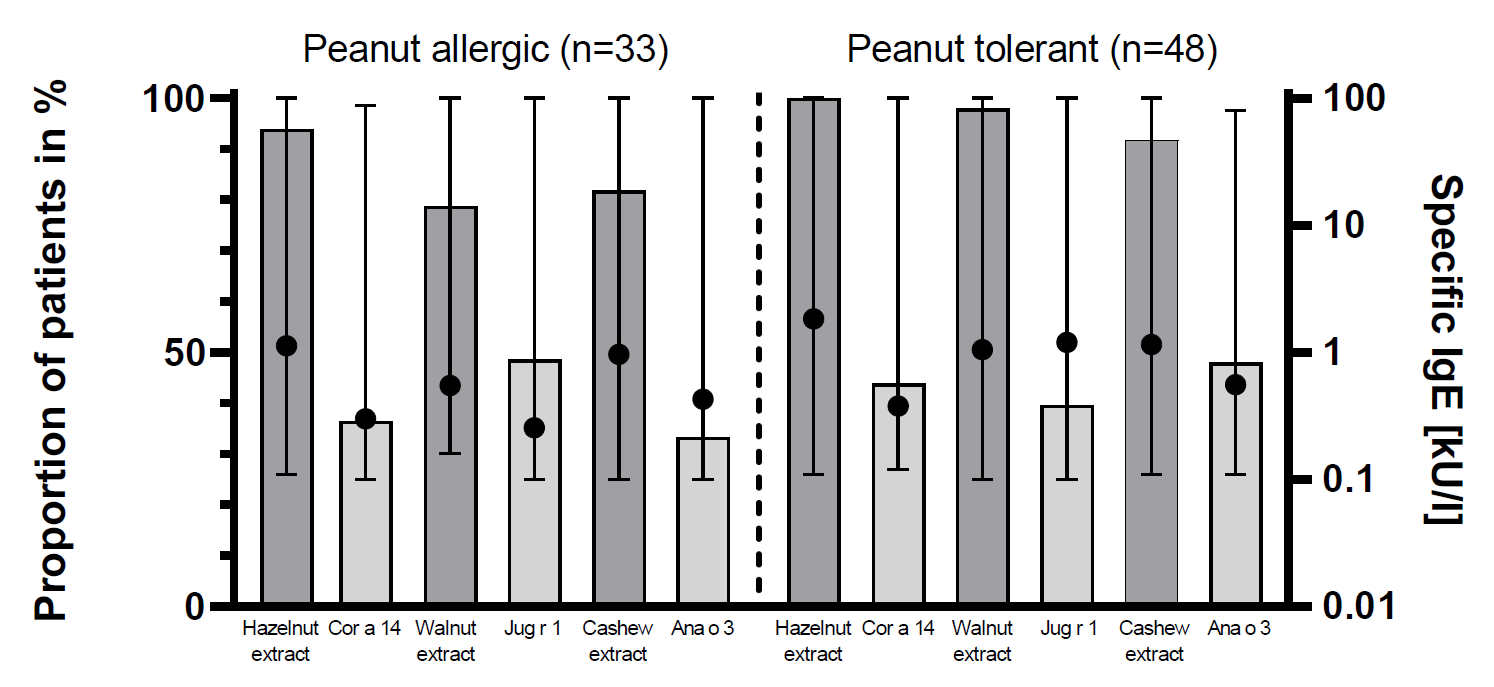


**Figure S1.** Figure S1 shows the proportion of peanut allergic (n=33) and peanut tolerant (n=48) patients being sensitized (specific IgE ≥ 0.1 kU/l) to hazelnut, walnut and cashew and to their corresponding 2S albumins Cor a 14, Ana o 3 and Jug r 1 as well as median and range of the corresponding specific IgE levels (clinical relevance of peanut sensitization was known in 81 out of 101 patients). There was no significant difference between peanut-allergic and peanut-tolerant children regarding their sensitization pattern to tree nuts.
